# Efficacy and Safety of Lenalidomide for Treatment of Low-/Intermediate-1-Risk Myelodysplastic Syndromes with or without 5q Deletion: A Systematic Review and Meta-Analysis

**DOI:** 10.1371/journal.pone.0165948

**Published:** 2016-11-08

**Authors:** Xin-yue Lian, Zhi-hui Zhang, Zhao-qun Deng, Pin-fang He, Dong-ming Yao, Zi-jun Xu, Xiang-mei Wen, Lei Yang, Jiang Lin, Jun Qian

**Affiliations:** 1 Department of Hematology, Affiliated People’s Hospital of Jiangsu University, Zhenjiang, Jiangsu, People’s Republic of China; 2 Laboratory Center, Affiliated People’s Hospital of Jiangsu University, Zhenjiang, Jiangsu, People’s Republic of China; University of Maryland Baltimore County, UNITED STATES

## Abstract

**Background:**

Lenalidomide could effectively induce red blood cell (RBC) transfusion independence (TI) in patients with lower-risk (Low/Intermediate-1) myelodysplastic syndrome (MDS) with or without 5q deletion. However whether lenalidomide ultimately improves the overall survival (OS) of lower-risk MDS patients and reduces the progression to AML remains controversial.

**Method:**

A meta-analysis was conducted to examine the efficacy and safety of lenalidomide in the treatment of lower-risk MDS. Efficacy was assessed according to erythroid hematologic response (HI-E), cytogenetic response (CyR), OS and AML progression. Safety was evaluated based on the occurrence rates of grades 3–4 adverse events (AEs).

**Results:**

Seventeen studies were included consisting of a total of 2160 patients. The analysis indicated that the overall rate of HI-E was 58% with 95% confidence interval (CI) of 43–74%. The pooled estimates for the rates of CyR, complete CyR, and partial CyR were 44% (95% CI 19–68%), 21% (95% CI 13–30%) and 23% (95% CI 15–32%), respectively. The patients with 5q deletion had significantly higher rate of HI-E and CyR than those without 5q deletion (P = 0.002 and 0.001, respectively). The incidences of grades 3–4 neutropenia, thrombocytopenia, leukopenia, anemia, deep vein thrombosis, diarrhea, fatigue and rash were 51% (95% CI 30–73%), 31% (95% CI 20–42%), 9% (95% CI 5–13%), 7% (95% CI 2–12%), 3% (95% CI 2–5%), 3% (95% CI 1–5%), 2% (95% CI 1–4%) and 2% (95% CI 1–3%), respectively. Lenalidomide significantly improved OS (HR: 0.62, 95% CI 0.47–0.83, *P* = 0.001) and lowered the risk of AML progression in del(5q) patients (RR: 0.61, 95% CI 0.41–0.91, *P* = 0.014).

**Conclusions:**

In spite of the AEs, lenalidomide could be effectively and safely used for the treatment of lower-risk MDS patients with or without 5q deletion.

## 1. Introduction

Myelodysplastic syndromes (MDS) include a group of hematopoietic stem cell disorders characterized by dysplastic changes, peripheral blood cytopenia and progression to acute myeloid leukemia (AML) [1–7]. At present, allogenetic stem cell transplantation (SCT) has become the only potentially curative therapy. However, it is applied in only a minority of patients owing to concomitant comorbidities and limited availability of donor sources. Therefore, novel pharmacologic therapies capable of relieving early symptoms or delaying disease progression are anticipated.

Lenalidomide (Revlimid), a thalidomide analogue, is an immunomodulatory agent with multiple mechanisms of action, which are deemed to contain the direct targeting of MDS clones, immunomodulation and erythropoiesis restoration [8–11]. Recently, lenalidomide has been approved for the treatment of lower-risk (Low/Intermediate-1) MDS patients with 5q deletion in USA and several other countries. In addition, previous studies suggested that lenalidomide is also active in patients without 5q deletion. Trials of studying the role of lenalidomide in lower-risk MDS have shown that lenalidomide effectively induces red blood cell (RBC) transfusion independence (TI) in lower-risk MDS patients with or without 5q deletion. Evidences are provided by previous studies that the treatment with lenalidomide is associated with an extension of overall survival (OS) and a decreased risk of AML progression. However, some studies reported that patients with MDS who fail to achieve erythroid or cytogenetic remission after the treatment with lenalidomide have an increased risk of AML progression. Consequently, whether lenalidomide ultimately improves the OS of lower-risk MDS patients and resists the progression to AML remains controversial.

This systematic review and meta-analysis aimed to evaluate the efficacy and safety of lenalidomide for the treatment of lower-risk MDS patients with or without 5q deletion.

## 2. Materials and Methods

### 2.1 Search strategy

The pertinent studies were obtained by searching Pubmed, Embase and Cochrane Library (Cochrane Central Register of Controlled Trials) up to March 2016. Electronic searches were performed combining MeSH terms and free words using the following search algorithm: ((myelodysplastic syndrome) OR (dysmyelopoietic syndrome) OR (MDS) AND (lenalidomide) OR (revlimid) OR (CC5013)). The publication year was not restricted. However, the publication language of studies was limited to English. Any disagreement in processes was resolved by a third reviewer.

### 2.2 Study selection and Endpoints

Studies meeting the following criteria were included: 1) the studies focused on both prospective and retrospective studies of lenalidomide for the treatment of lower-risk MDS; 2) the studies provided sufficient information, like the number of patients in each group or incidences of side effects. Studies were excluded if they did not meet the above-mentioned requirements or data insufficient for extraction. Reviews and case reports were also excluded. Any divergence among reviewers was discussed and reached consensus finally. Our efficacy endpoints of interest included erythroid hematologic response (HI-E), cytogenetic response (CyR), complete CyR (CCyR), partial CyR (PCyR), OS and proportion of AML progression. The safety endpoint was treatment-related grades 3–4 adverse events (AEs).

### 2.3 Data extraction

Two investigators extracted data from each eligible article independently and blindly according to standard data extraction forms. The following information was obtained from each article: first author, publication year, study population, patient demographic characteristics (age, gender, etc.), intervention treatment (drug name, dose and administration routine), outcome measures and study results.

When more than one publication of the same study existed, only the publication with the most complete data was extracted in the analysis. Disagreements were resolved by inquiring a third investigator.

### 2.4 Quality assessment

The quality of the enrolled articles was assessed by two authors according to the Cochrane Collaboration Reviewers’ Handbook for Systematic Reviews of Interventions. A quality review involved random sequence generation (selective bias); allocation concealment (selective bias); blinding of participants and personnel (performance bias); blinding of outcomes assessment (detection bias); incomplete outcome data (attrition bias); selective reporting (reporting bias); and other bias [12–13]. Based on the quality assessment criteria, each article was divided into three different categories. If all criteria were met, we ranked it as class A; if more than one criterion was not reached or was unclear, we ranked it as class B; the assessment also suggested that if no one reached criteria, we ranked it as class C. Mutual consensuses were reached by discussion if any disagreement put forward.

### 2.5 Statistical analysis

Statistical analysis was conducted using Stata 11.0 software (Stata Corporation, College Station, TX, USA). We calculated estimated proportions (ES) with 95% confidence intervals (CIs) for ratio outcomes. Hazard ratio (HR), odds ratio (OR) and relative risk (RR) with 95% CIs were calculated for dichotomous outcomes. Statistical heterogeneity across studies was analysed using the χ^2^ test and I^2^ statistic. An I^2^ statistic < 50.0% and *P* ≥ 0.10 suggested that low heterogeneity existed. If I^2^ statistic was more than 50.0% and *P* < 0.10, it was believed to have significant statistical heterogeneity in included studies. If I^2^ < 50.0% and *P* ≥ 0.10, we chose a fixed-effect model. Otherwise, a random-effect model was used. Statistical significance was defined at *P* values of less than 0.05. Finally, funnel plots were visually assessed in order to evaluate potential publication bias. At the same time, Begg’s test and Egger’s test were also statistically conducted.

## 3. Results

### 3.1 Characteristics of studies

**Fig 1:**
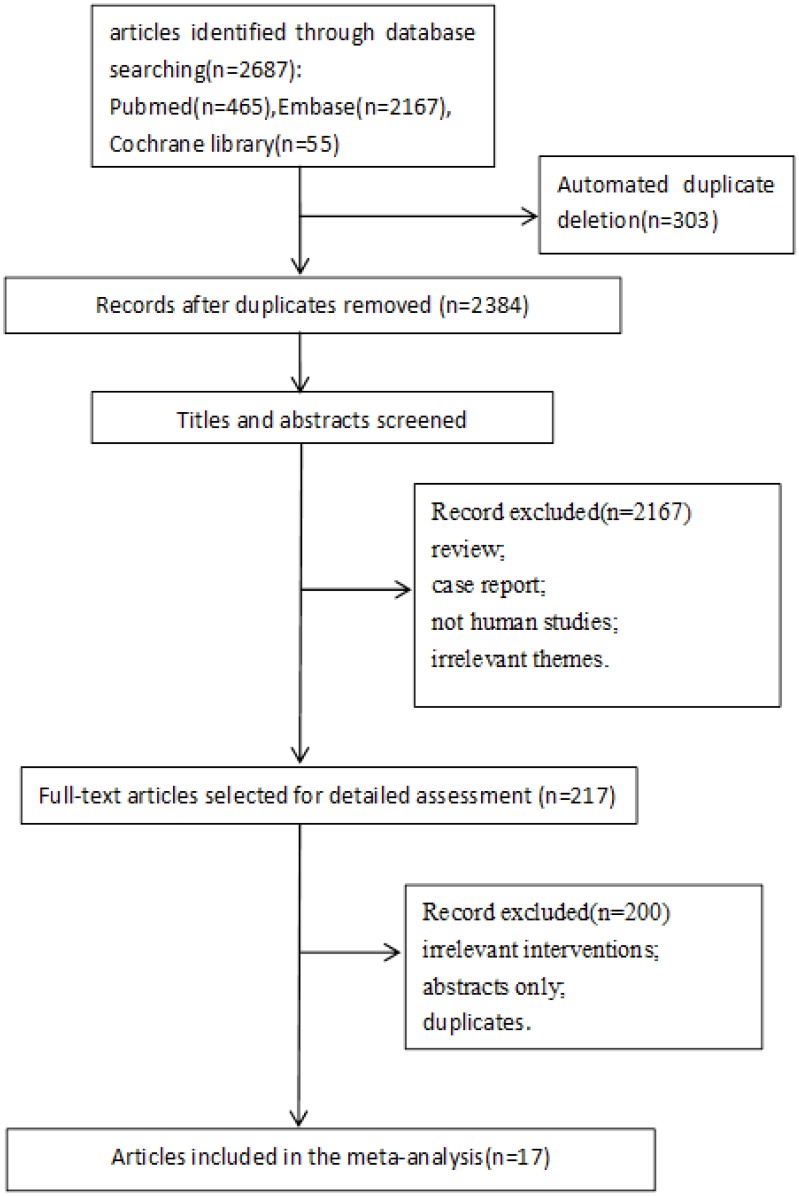
PRISMA flowchart describing the study’s systematic literature search and study selection.

A comprehensive literature search was conducted. The initial search yielded 2687 articles, of which 17 articles containing 6 randomized controlled trials (RCTs) were finally included in the present meta-analysis [14–30]. The detailed selection process was presented in Fig 1, and the characteristics of the included studies were described in Table 1. The cytogenetic features at baseline in del(5q) and non-del(5q) patients were described in the Tables 2 and 3. In all, twelve studies analysed HI-E, eight studies assessed CyR, three studies evaluated OS, four studies appraised AML progression, and ten studies analysed the grades 3–4 AEs.

**Table 1:** Characteristics of the studies included.

Study	No. of patients	Age range(year)	5q-	Treatment(mg/day)	HI-E rates	No. of patients with cytogenetic abnormalities	CyR rates	OS(median,months)
Fenaux(2011)^[^^14^^]^	47	40–86	+	Len 5	NA	47	0.25	35.5
	41	36–84	+	Len 10	NA	41	0.5	36.9
	51	39–85	+	Placebo	NA	51	0	35.9
Komrokji(2012)^[^^15^^]^	39	45–85	+/-(-:32)	Len 10	0.307	39	NA	NA
Adès(2012)^[^^16^^]^	71	69.8(median)	+	Len 10	NA	71	NA	NA
	71	71.3(median)	+	ESA or thalidomide	NA	71	NA	NA
Kuendgen(2013)^[^^17^^]^	295	32–94	+	Len 5 or 10	NA	293	NA	62
	125	30–91	+	SC	NA	125	NA	44
List(2006)^[^^18^^]^	148	37–95	+	Len 10	0.757	85	0.729	NA
Sánchez-García(2014)^[^^19^^]^	86	35–90	+	Len 5 or 10	NA	86	0.592	62
	129	37–90	+	SC	NA	129	NA	53
Abouyahya(2013)^[^^20^^]^	19	53–85	+	Len 5 or 10	0.579	19	NA	NR(patients with HI-E)
								13.7(patients without HI-E)
Oliva(2013)^[^^21^^]^	45	63–81	+	Len 10	0.822	45	0.644	NA
Butrym(2015)^[^^26^^]^	36	25–83	+	Len 10	0.917	33	NA	NA
Arcioni(2015)^[^^28^^]^	190	75(median)	+	Len 10	0.858	190	0.026	NA
Arrizabalaga(2013)^[^^29^^]^	20	41–85	+/-(-:5)	Len 10	0.65	20	NA	NA
Le Bras(2009)^[^^30^^]^	95	42–92	+	Len 10	0.65	95	0.6	95
Zeidan(2015)^[^^22^^]^	37	66.3	-	Len 10	0.38	NA	NA	104
Toma(2016)^[^^23^^]^	131	64–76	-	Len 10	0.313	131	NA	NA
Sibon(2012)^[^^24^^]^	31	42–87	-	Len 5 or 10	0.484	31	NA	NA
Raza(2008)^[^^25^^]^	214	27–94	-	Len 10	0.327	207	0.19	NA
Santini(2014)^[^^27^^]^	160	46–87	-	Len 10	NA	159	0.33	NR
	79	43–87	-	Placebo	NA	79	0	NR

Abbreviations: HI-E: erythroid hematologic response; OS: overall survival; NA: not available; NR: not reached; +: del(5q); -: non-del(5q); ESA: Erythropoiesis-stimulating agents; SC: supportive care.

**Table 2:** Cytogenetic features at baseline in del(5q) patients.

Study	No. of patients with cytogenetic abnormalities	No. of patients with isolated del(5q)	No. of patients with del(5q) plus ≥ 1 additional abnormality
Fenaux(2011)^[^^14^^]^	139	106	33
Komrokji(2012)^[^^15^^]^	39	NA	NA
Adès(2012)^[^^16^^]^	142	114	28
Kuendgen(2013)^[^^17^^]^	293	224	69
List(2006)^[^^18^^]^	85	64	21
Sánchez-García(2014)^[^^19^^]^	215	169	46
Abouyahya(2013)^[^^20^^]^	19	13	6
Oliva(2013)^[^^21^^]^	45	34	11
Butrym(2015)^[^^26^^]^	33	NA	NA
Arcioni(2015)^[^^28^^]^	190	NA	NA
Arrizabalaga(2013)^[^^29^^]^	20	NA	NA
Le Bras(2009)^[^^30^^]^	95	NA	NA

**Table 3:** Cytogenetic features at baseline in non-del(5q) patients.

Study	No. of patients with cytogenetic abnormalities	No. of patients with favorable cytogenetics	No. of patients with intermediate cytogenetics	No. of patients with unfavorable cytogenetics
Toma(2016)^[^^23^^]^	131	110	21	0
Sibon(2012)^[^^24^^]^	31	26	3	2
Raza(2008)^[^^25^^]^	207	177	27	3
Santini (2014)^[^^27^^]^	238	197	41	0
Zeidan(2015)^[^^22^^]^	NA	NA	NA	NA

NA: not available

### 3.2 Quality of the individual studies

Quality assessment of the seventeen studies showed that the levels of quality of all studies included were classified as B. The results of all the risk of bias assessment were summarized in S2 Table.

### 3.3 Efficacy

#### 3.3.1 Erythroid response

**Fig 2:**
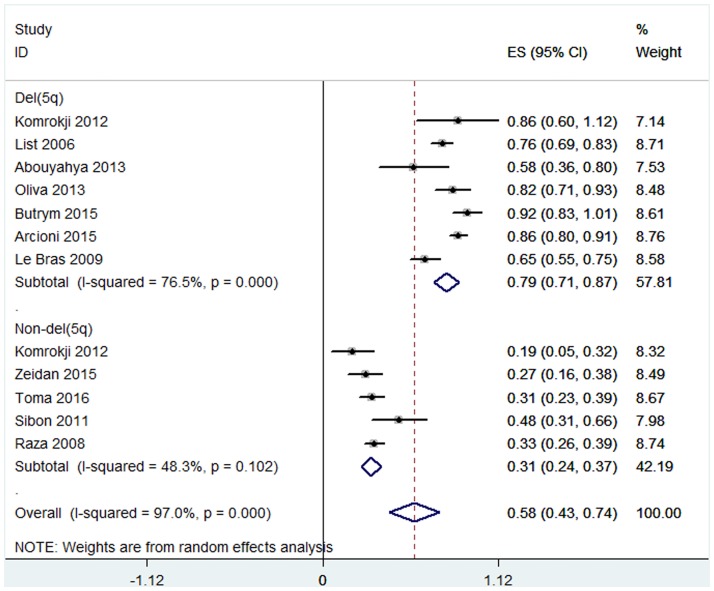
Forest plot of the estimated proportions (95% confidence interval) for HI-E in MDS patients treated with lenalidomide.

**Fig 3:**
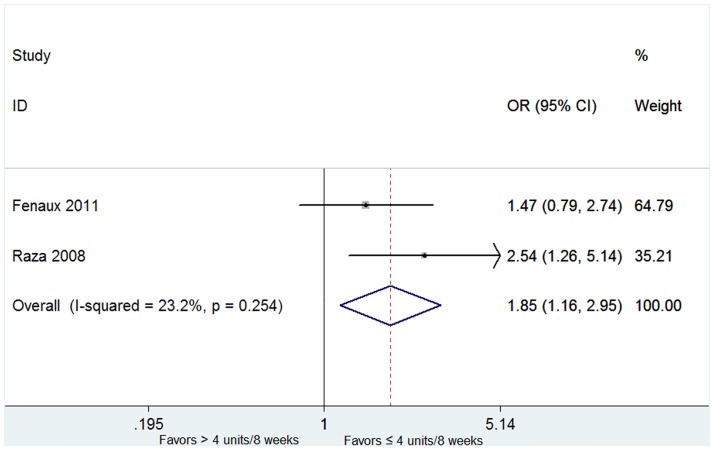
Forest plot of the odds ratio for RBC-TI in patients with transfusion burden ≤ 4 units/8 weeks vs > 4 units/8 weeks.

**Fig 4:**
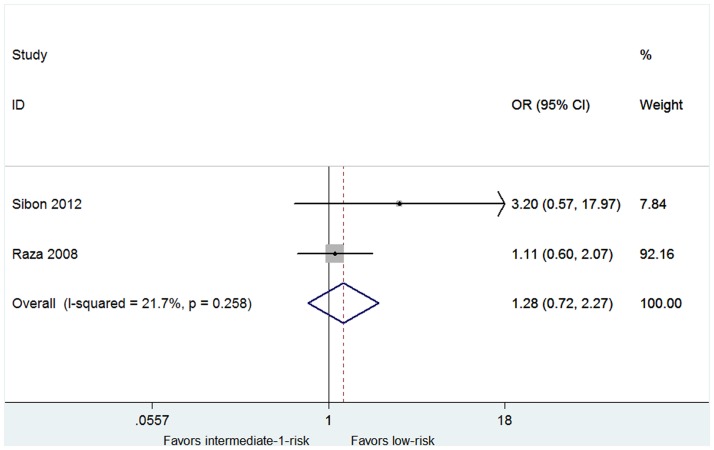
Forest plot of the odds ratio for achievement of RBC-TI in patients with low-risk vs intermediate-1-risk.

**Fig 5:**
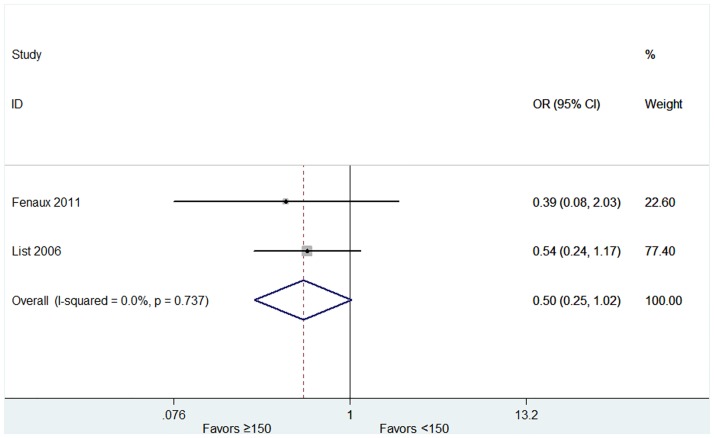
Forest plot of the odds ratio for achievement of RBC-TI in patients with platelet count ≥ 150 × 10^9^/L vs < 150 × 10^9^/L.

The overall rates of HI-E were presented in twelve articles [15,18,20–26,28–30]. High heterogeneity of the overall rate of HI-E was found from the data (*P* < 0.001, I^2^ = 97.0%), and thus the random-effect model was chosen. The pooled estimates for the overall rate of HI-E was 58% (95% CI 43–74%, Fig 2). The patients with 5q deletion had a significantly higher rate of HI-E (79%, 95% CI 71–87%) than that without 5q deletion (31%, 95% CI 24–37%) (P = 0.002, Fig 2). No significant difference was found in the rate of RBC-TI in patients with favorable or unfavorable karyotypes (S3 File). In both del(5q) and non-del(5q) patients, transfusion burden ≤ 4 units/8 weeks was associated with a higher rate of RBC-TI (Fig 3). The proportion of the RBC-TI rate in low-risk non-del(5q) patients was higher than those in intermediate-1-risk non-del(5q) patients (Fig 4). Similarly, platelet count ≥ 150 × 10^9^/L was associated with a higher rate of RBC-TI in del(5q) patients (Fig 5). It was difficult to evaluate whether neutropenia and increased marrow blasts had significant impact on the treatment of lenalidomide because the data involved with these parameters was not abundant to analyse.

#### 3.3.2 Cytogenetic response

**Fig 6:**
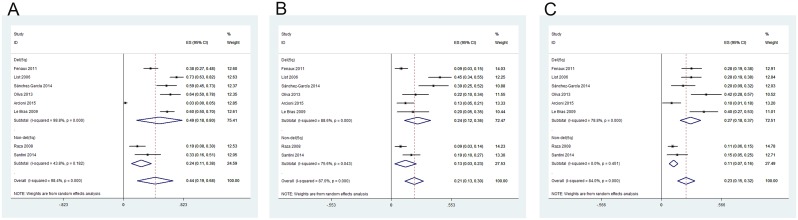
Forest plot of the estimated proportions (95% confidence interval) for (A) cytogenetic response (CyR) in MDS patients treated with lenalidomide; (B) complete cytogenetic response (CCyR); (C) partial cytogenetic response (PCyR).

Eight studied described the rate of CyR in lower-risk MDS patients with or without 5q deletion [14,18,19,21,25,27,28,30]. The heterogeneity test indicated that significant heterogeneity existed, hence the random-effect model was chosen. The pooled estimates for the rates of CyR, CCyR, and PCyR were 44% (95% CI 19–68%), 21% (95% CI 13–30%) and 23% (95% CI 15–32%), respectively (Fig 6). The rates of CyR, CCyR and PCyR for patients with 5q deletion were significantly higher than those in non-del(5q) patients (P = 0.001, <0.001 and = 0.011, respectively) (Fig 6).

#### 3.3.3 Overall Survival

**Fig 7:**
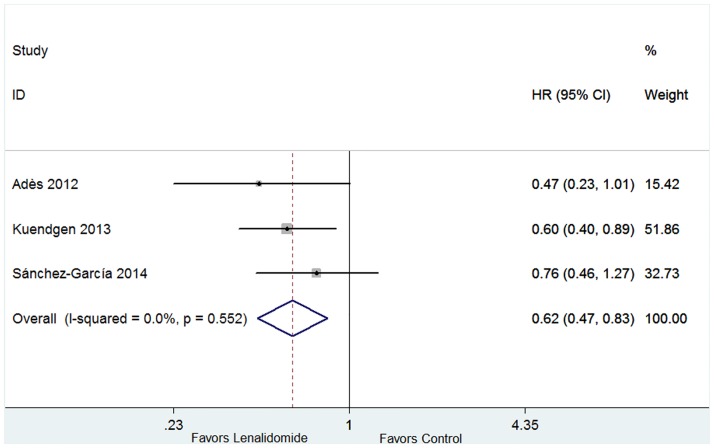
Forest plot of the hazard ratio for overall survival (OS) in lower-risk MDS patients with 5q deletion.

Three trials which included control group (placebo, erythropoiesis-stimulating agents or thalidomide) contributed to OS analysis [16,17,19]. The heterogeneity test indicated that no significant heterogeneity existed (*P* = 0.552, I^2^ = 0.0%), and hence the fixed-effect was used. The pooled estimate of the HR was 0.62 (95% CI 0.47–0.83, P = 0.001; Fig 7), suggesting that lenalidomide could prolong the OS of lower-risk MDS patients with 5q deletion.

#### 3.3.4 AML progression

**Fig 8:**
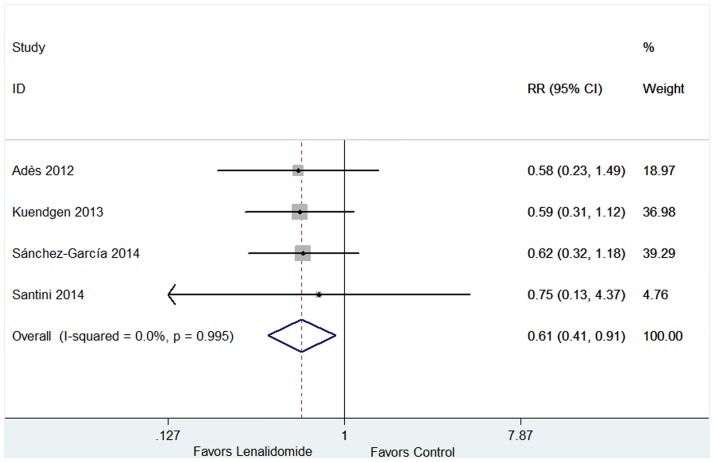
Forest plot of the relative risks for AML progression in case group (lenalidomide-treated) and control group (lenalidomide-untreated).

**Fig 9:**
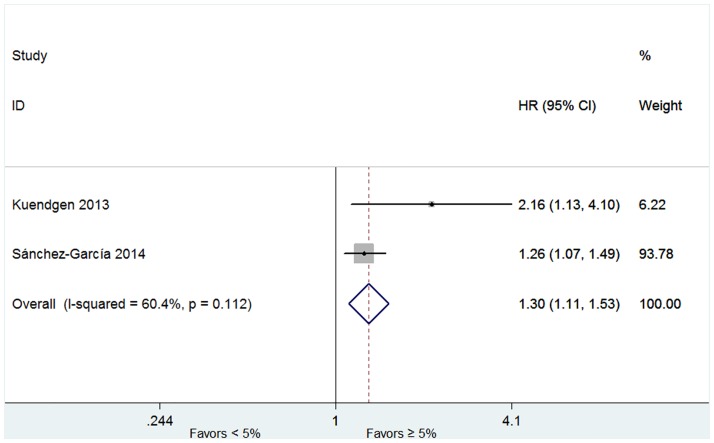
Forest plot of the hazard ratio for AML progression in patients with bone marrow blasts ≥ 5% or < 5%.

**Fig 10:**
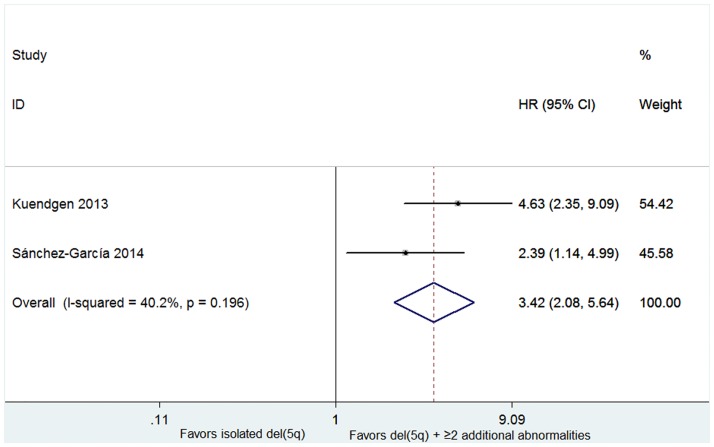
Forest plot of the hazard ratio for AML progression in patients with isolated del(5q) and del(5q) plus ≥ 2 additional abonormalities.

Four studies including 762 patients reported the probabilities of progression to AML in patients with or without lenalidomide treatment [16,17,19,27]. No significant heterogeneity for probabilities was found (*P* = 0.995, I^2^ = 0.0%), and therefore the fixed-effect was utilized. The results indicated that RR was 0.61 (95% CI 0.41–0.91, *P* = 0.014; Fig 8). The proportion of AML progression in lenalidomide-treated cohort was lower than the lenalidomide-untreated group. AML progression rates were higher in patients with an excess of marrow blasts (more than 5% blasts) (HR 1.30, 95% CI 1.11–1.53; P = 0.002; Fig 9) and in patients with del(5q) plus ≥ 2 additional abnormalities (HR 3.42, 95% CI 2.08–5.64; P < 0.001; Fig 10).

### 3.4 Safety

**Fig 11:**
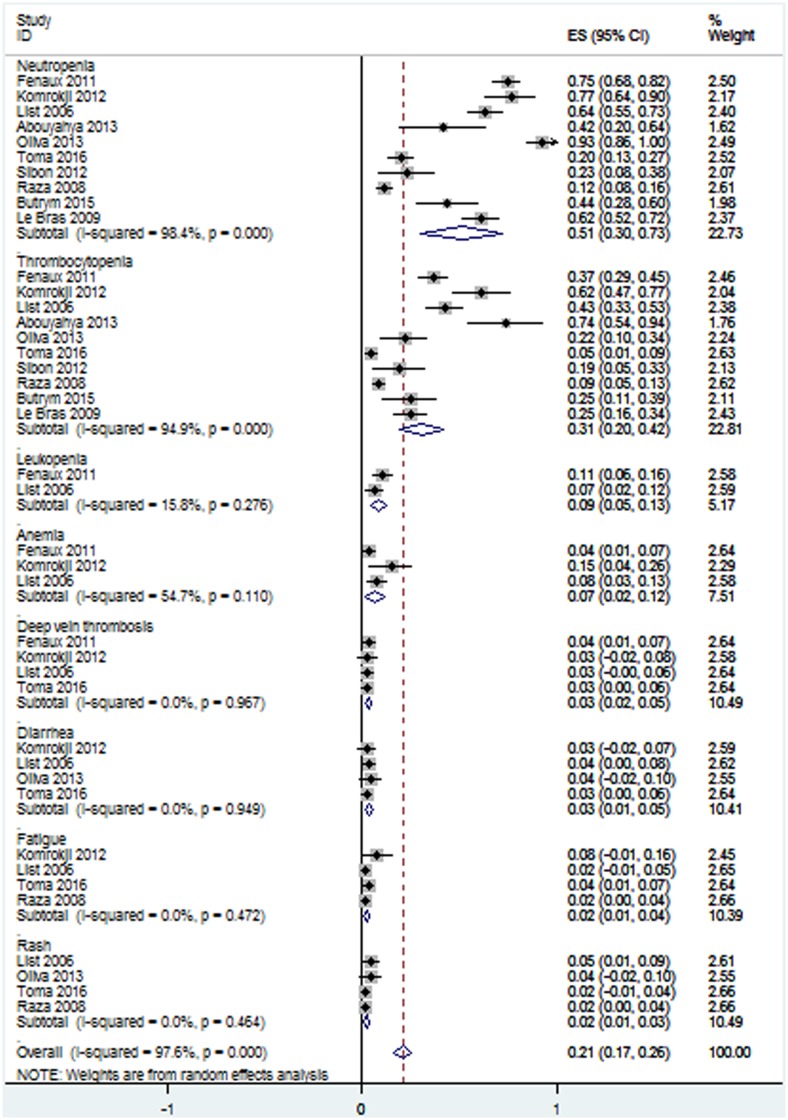
Forest plot of the estimated proportions of grades 3–4 adverse events.

**Fig 12:**
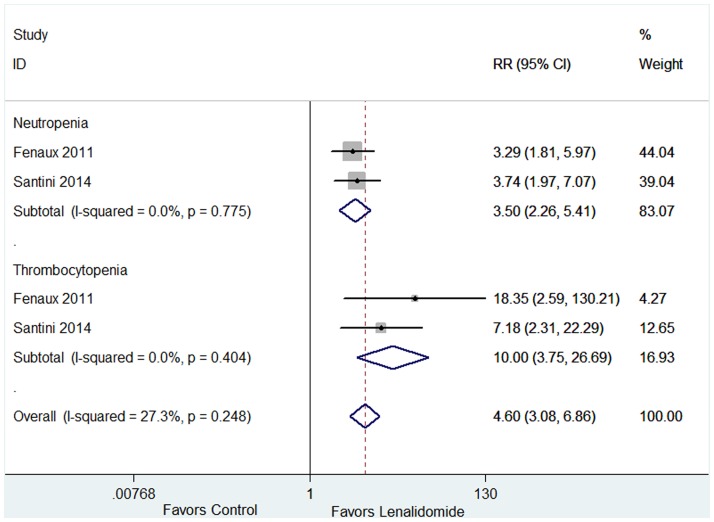
Forest plot of the relative risks of adverse events in case group (lenalidomide 5 or 10mg/day) and control group (placebo).

AEs of lenalidomide and relevant data were presented in ten articles [14,15,18,20,21,23–26,30]. All studies investigated the incidences of grades 3–4 AEs. The incidences of grades 3–4 AEs of the ten studies showed significant statistical heterogeneity (Fig 11), and thus the random-effect model was adopted. Leukopenia, anemia, deep vein thrombosis, diarrhea, fatigue and rash had no significant statistical heterogeneity (Fig 11), and thus we chose fixed-effect model. The analysis revealed that the pooled estimates for the incidences of neutropenia, thrombocytopenia, leukopenia, anemia, deep vein thrombosis, diarrhea, fatigue and rash were 51% (95% CI 30–73%), 31% (95% CI 20–42%), 9% (95% CI 5–13%), 7% (95% CI 2–12%), 3% (95% CI 2–5%), 3% (95% CI 1–5%), 2% (95% CI 1–4%) and 2% (95% CI 1–3%) respectively. Among the ten studies, there were two studies comparing AEs of lenalidomide group (Len = 5 mg/day or 10 mg/day) verus placebo group [14,27]. Lenalidomide-treated group had significantly higher incidences of neutropenia (*P* < 0.001) and thrombocytopenia (*P* < 0.001) (Fig 12).

### 3.5 Publication bias and Sensitivity analysis

To assess the potential publication bias, we inspected the funnel plots and Egger’s regression models. No asymmetrical distribution was found and no significant publication bias was observed. Sensitivity analyses were performed because of the included small-sample studies, the allele model indicated that no single study significantly influenced the pooled outcomes, suggesting the results of this meta-analysis were stable.

## 4. Discussion

This systematic review indicates that lenalidomide brings HI-E to lower-risk MDS patients. Although lenalidomide is useful to lower-risk MDS patients regardless of 5q deletion or not, the HI-E rate is significantly higher in 5q-deleted patients compared with that in patients without deletion 5q. Meanwhile, the treatment with lenalidomide yielded high rates of RBC-TI in transfusion-dependent patients with lower-risk del(5q) MDS [14,18,31,32]. Moreover, lenalidomide induces higher CyR in lower-risk MDS patients with 5q deletion. It is worthwhile to note that the decline of the platelet count was an important parameter related to a reduced probability of TI [18]. In addition, HI-E and CyRs induced by lenalidomide is potentially related to a longer OS and a reduced risk of progression to AML in 5q-deleted patients. Lenalidomide reduces del(5q) progenitors to induce CyR. However, it should be noted that lenalidomide could not eradicate del(5q) stem cells which contribute to the expansion of the del(5q) clone and clinical and cytogenetic progression [33,34].

Over the past decade, myelosuppression occurred commonly during lenalidomide therapy in patients with lower-risk MDS [17,25], and thus we assessed the occurrence rates of grades 3–4 AEs of lenalidomide used in lower-risk MDS patients. Based on the analysis of pooled data, the incidence rates of neutropenia and thrombocytopenia were obviously high and the RR of grades 3–4 neutropenia and thrombocytopenia was higher in the lenalidomide-treated group. The survival time was longer in the group of patients with grades 3–4 AEs because the erythroid and cytogenetic responses occurred after the treatment of lenalidomide[26]. Therefore, lenalidomide could be safely used to treat lower-risk MDS patients.

According to the results of this systematic review, transfusion burden ≤ 4 units/8 weeks was associated with a higher achievement of RBC-TI in both del(5q) and non-del(5q) patients. In del(5q) patients, platelet count ≥ 150 × 10^9^/L has an association with a higher rate of RBC-TI. The RBC-TI rate was also higher in low-risk non-del(5q) patients than intermediate-1-risk non-del(5q) patients. At the same time, AML progression rates were higher in del(5q) patients plus ≥ 2 additional abnormalities, and in patients with an excess of marrow blasts. In additon, older age, higher baseline ferritin levels, and higher transfusion burden were also related to an increases risk of AML progression [14]. Some studies found that patients without a continuous erythroid and cytogenetic remission after treatment had an increased risk of AML progression [31]. The relatively high incidence of AML progression was put down to the fact that the interval between the diagnosis of MDS and the onset of lenalidomide treatment was particularly long, resulting in a longer history of RBC transfusions. Furthermore, genetic instability and clonal evolution appear to be the driving forces of AML progression in MDS patients who fail to achieve continuous erythroid and cytogenetic remission after treatement with lenalidomide [21]. Therefore, we should strengthen the monitoring of morphological and cytogenetic follow-up and consider to stop using lenalidomide when genetic instability and clonal evolution are detected especially in patients who fail to achieve continuous remission after therapy.

Although lenalidomide can be effectively used to treat lower-risk MDS, not all patients respond to the treatment. Thus, it is important to identify who are suitable for lenalidomide treatment. Some studies have focused on the relationship between gene mutations and outcomes. In lower-risk MDS patients with 5q deletion who received lenalidomide treatment, unmutated TP53 status showed a trend to predict the achievement of HI and CyR [35]. The presence of a DNMT3A mutation could be predictive of better responses of lower-risk MDS patients without 5q deletion to the lenalidomide treatment. Especially, it was independently related to HI-E [36]. Further prospective studies are mandatory to predict which MDS patients may respond to lenalidomide. Some other studies suggested that a number of somatic mutations including SF3B1, TET2 and ASXL1 were associated with the poor prognosis. Consequently, the gene-targeted therapy could be taken into consideration to combine with the lenalidomide treatment.

Several limitations of this meta-analysis should be acknowledged. First, the limited number of included studies influenced the power of our analysis. As such, the data were not robust enough to compare the safety of lenalidomide with other therapies. Second, our analysis is limited by the high heterogeneity of some included studies. Third, a host of trials were not randomized controlled sutdies. Therefore, we can only analyse the estimated proportions of outcomes. Fourth, limited randomised trials are available to analyse the HR of the OS, especially the patients without 5q deletion. Fewer studies are concerned with the lenalidomide therapy in non-del(5q) patients. Therefore, the comparison between del(5q) patients and non-del(5q) patients may have deviations.

## 5. Conclusions

This systematic review and meta-analysis indicated that lenalidomide is active for lower-risk MDS patients with or without 5q deletion not only in reducing RBC transfusion burden and yielding CyR, but also in extension of survival and a reduction in the risk of AML progression. Although lenalidomide could be safely used to treat lower-risk MDS patients, neutropenia, thrombocytopenia and leukopenia should be watched closely for patients undergoing the treatment of lenalidomide.

## Supporting Information

S1 File Forest plot of the estimated proportions (95% confidence interval) for HI-E stratified by type of prospective and retrospective studies. (TIF)

S2 File Forest plot of the estimated proportions (95% confidence interval) for CyR stratified by type of prospective and retrospective studies. (TIF)

S3 File Forest plot of the odds ratio for achievement of RBC-TI in patients with favorable kayotype vs unfavorable kayotype. (TIF)

S4 File Search strategy. (DOCX)

S1 Table PRISMA Checklist. (DOC)

S2 Table Risk of bias summary. (DOCX)
