# Supplementary figures and images for: Efficacy and Safety of Lenalidomide for Treatment of Low-/Intermediate-1-Risk Myelodysplastic Syndromes with or without 5q Deletion: A Systematic Review and Meta-Analysis
Source: PLoS One. 2016 Nov 8;11(11):e0165948. doi: 10.1371/journal.pone.0165948 (PMC5100926; doi:10.1371/journal.pone.0165948)

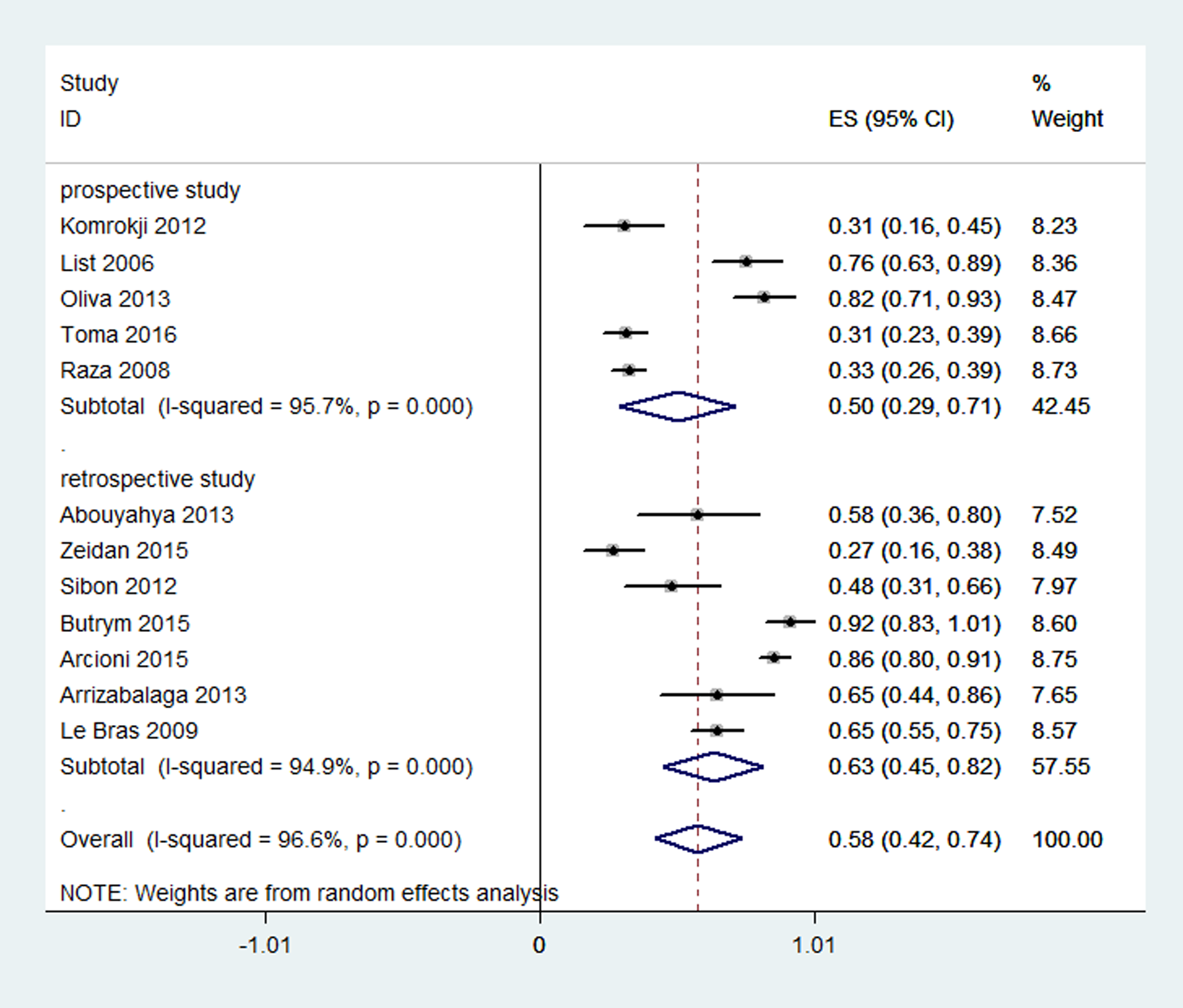

Supplement: S1 File — (TIF) [file pone.0165948.s001.tif]

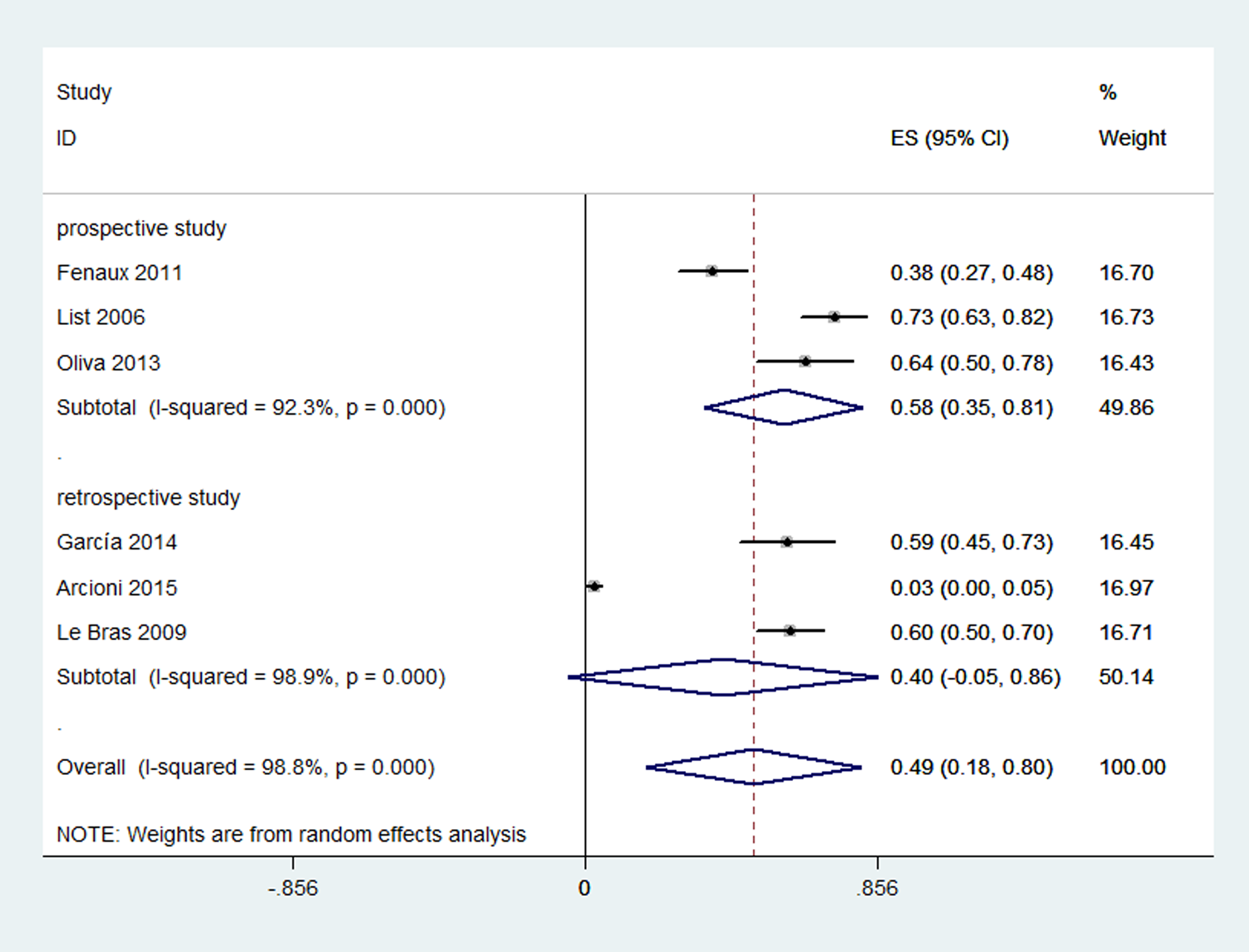

Supplement: S2 File — (TIF) [file pone.0165948.s002.tif]

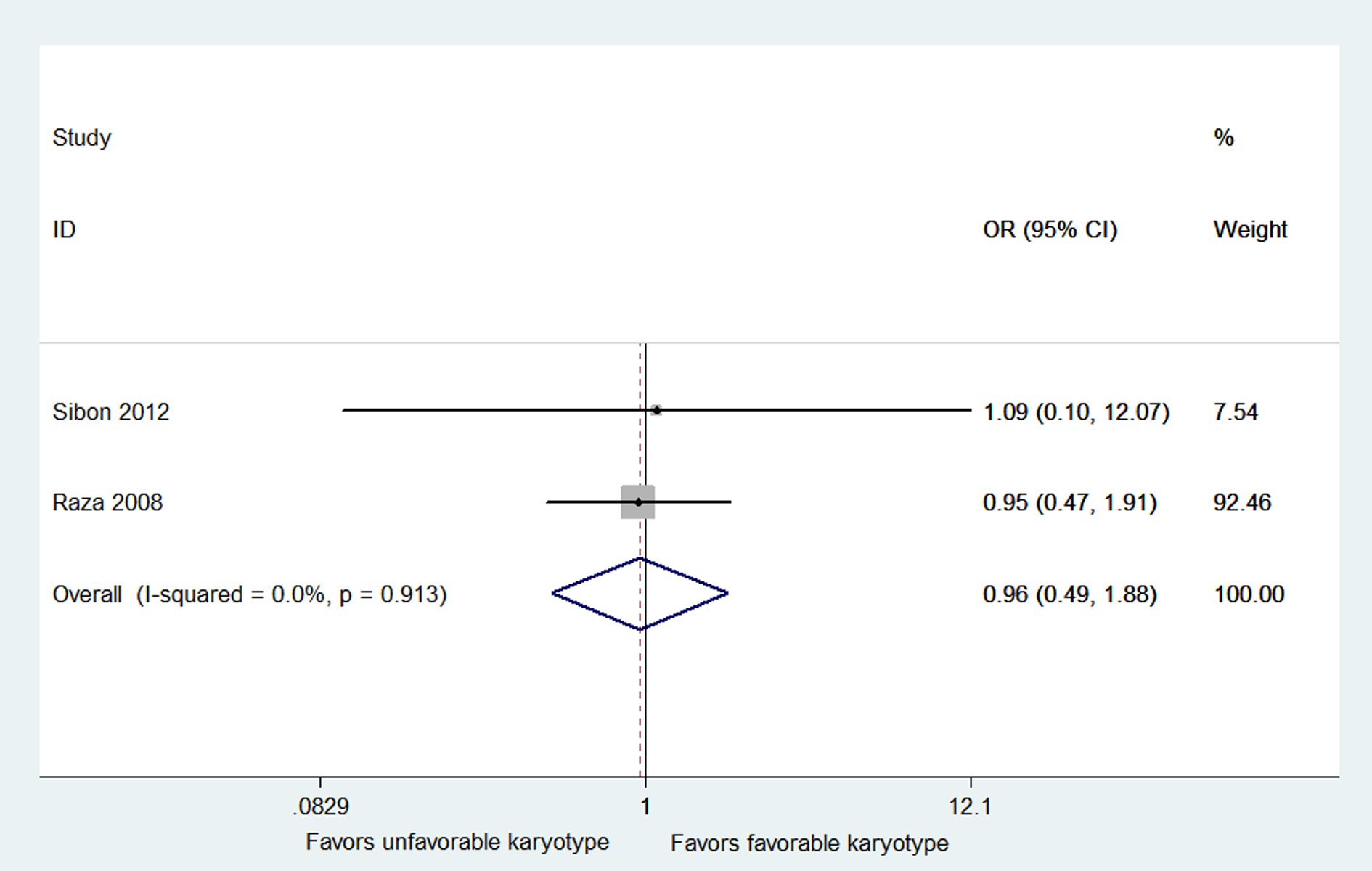

Supplement: S3 File — (TIF) [file pone.0165948.s003.tif]
