# Supplementary material for: Efficacy and Safety of Lenalidomide for Treatment of Low-/Intermediate-1-Risk Myelodysplastic Syndromes with or without 5q Deletion: A Systematic Review and Meta-Analysis
Source: PLoS One. 2016 Nov 8;11(11):e0165948. doi: 10.1371/journal.pone.0165948 (PMC5100926; doi:10.1371/journal.pone.0165948)
Supplement: S4 File — (DOCX) [file pone.0165948.s004.docx]

| Database | Strategy | Number |
| --- | --- | --- |
| Pubmed | ((("Myelodysplastic Syndromes"[MeSH]) OR ((Dysmyelopoietic Syndromes[Title/Abstract]) OR MDS[Title/Abstract]))) AND (("lenalidomide" [Supplementary Concept]) OR ((Revlimid[Title/Abstract]) OR CC5013[Title/Abstract])) | 465 |
| Cochrane Library | #1 MeSH descriptor: [Myelodysplastic Syndromes] explode all trees  #2 Dysmyelopoietic Syndromes: ti, ab,kw (Word variations have been searched)  #3 MDS: ti, ab,kw (Word variations have been searched)  #4 Myelodysplastic: ti, ab,kw (Word variations have been searched)  #5 Dysmyelopoietic: ti, ab,kw (Word variations have been searched)#5 aplastic anemia  #6 Syndromes: ti, ab,kw (Word variations have been searched)  #7 #4 OR #5  #8 #6 AND #7  #9 #7 OR #1 OR #2 OR #3  #10 Lenalidomide: ti, ab,kw (Word variations have been searched)  #11 Revlimid: ti, ab,kw (Word variations have been searched)  #12 CC5013: ti, ab,kw (Word variations have been searched)  #13 #10 OR #11 OR #12  #14 #9 AND #13 | 55 |
| EMBASE | #1 ‘myelodysplastic Syndromes’:ab,ti  #2 ‘dysmyelopoietic Syndromes’:ab,ti  #3 ‘MDS’ ’:ab,ti  #4 #1 OR #2 OR #3  #5 ‘lenalidomide’/exp  #6 ‘revlimid’/exp  #7 ‘CC5013’/exp  #8 #5 OR #6 OR #7  #9 #4 AND #8 | 2167 |
