# Supplementary material for: Efficacy and Safety of Lenalidomide for Treatment of Low-/Intermediate-1-Risk Myelodysplastic Syndromes with or without 5q Deletion: A Systematic Review and Meta-Analysis
Source: PLoS One. 2016 Nov 8;11(11):e0165948. doi: 10.1371/journal.pone.0165948 (PMC5100926; doi:10.1371/journal.pone.0165948)
Supplement: S2 Table — (DOCX) [file pone.0165948.s006.docx]

S2 Table. **Risk of bias summary**

| Study | Random sequence generation | Allocation concealment | Blinding of participants and personnel | Blinding of outcome assessment | Incomplete outcome data | Selective reporting | Other bias |
| --- | --- | --- | --- | --- | --- | --- | --- |
|  | selection bias | selection bias | performance bias | detection bias | attrition bias | reporting bias |  |
| Fenaux P(2011)^[14]^ | + | + | + | + | + | + | - |
| Komorkji RS(2012)^[15]^ | + | + | + | + | + | + | - |
| Adès L(2012)^[16]^ | - | - | + | + | + | + | - |
| Kuendgen A(2013)^[17]^ | - | - | + | + | - | + | - |
| List A(2006)^[18]^ | + | + | + | + | + | + | - |
| Sánchez-García J (2014)^[19]^ | - | - | - | + | - | + | - |
| Abouyahya I(2013)^[20]^ | - | - | - | + | - | + | - |
| Oliva EN(2013)^[21]^ | - | - | - | + | - | + | - |
| Zeidan AM(2015)^[22]^ | + | + | + | + | + | + | - |
| Toma A(2016)^[23]^ | + | + | + | + | + | + | - |
| Sibon D(2012)^[24]^ | - | - | - | + | - | + | - |
| Raza A(2008)^[25]^ | - | - | - | + | - | + | - |
| Butrym A(2015)^[26]^ | - | - | - | + | - | + | - |
| Santini V(2014)^[27]^ | + | + | + | + | + | + | - |
| Arcioni F(2015)^[28]^ | - | - | + | + | + | + | - |
| Arrizabalaga B(2013)^[29]^ | - | - | - | + | + | + | - |
| Le Bras F(2009)^[30]^ | - | - | + | + | + | + | - |
